# Meandering Main Pancreatic Duct as a Relevant Factor to the Onset of Idiopathic Recurrent Acute Pancreatitis

**DOI:** 10.1371/journal.pone.0037652

**Published:** 2012-05-24

**Authors:** Wataru Gonoi, Hiroyuki Akai, Kazuchika Hagiwara, Masaaki Akahane, Naoto Hayashi, Eriko Maeda, Takeharu Yoshikawa, Shigeru Kiryu, Minoru Tada, Kansei Uno, Hiroshi Ohtsu, Naoki Okura, Kazuhiko Koike, Kuni Ohtomo

**Affiliations:** 1 Department of Radiology, Graduate School of Medicine, The University of Tokyo, Bunkyo-ku, Tokyo, Japan; 2 Department of Computational Diagnostic Radiology and Preventive Medicine, The University of Tokyo Hospital, Bunkyo-ku, Tokyo, Japan; 3 Department of Radiology, The Institute of Medical Science, The University of Tokyo, Minato-ku, Tokyo, Japan; 4 Department of Gastroenterology, Graduate School of Medicine, The University of Tokyo, Bunkyo-ku, Tokyo, Japan; 5 Department of Clinical Bioinformatics, Graduate School of Medicine, The University of Tokyo, Bunkyo-ku, Tokyo, Japan; 6 Department of Radiology, Japan Labour Health and Welfare Organization, Kanto Rosai Hospital, Kawasaki City, Kanagawa, Japan; Technische Universität München, Germany

## Abstract

**Background:**

Meandering main pancreatic duct (MMPD), which comprises loop type and reverse-Z type main pancreatic duct (MPD), has long been discussed its relation to pancreatitis. However, no previous study has investigated its clinical significance. We aimed to determine the non-biased prevalence and the effect of MMPD on idiopathic pancreatitis using non-invasive magnetic resonance (MR) technique.

**Methods and Findings:**

A cross-sectional study performed in a tertiary referral center. The study enrolled 504 subjects from the community and 30 patients with idiopathic pancreatitis (7 acute, 13 chronic, and 10 recurrent acute). All subjects underwent MR scanning and medical examination. MMPD was diagnosed when the MPD in the head of pancreas formed two or more extrema in the horizontal direction on coronal images of MR cholangiopancreatography, making a loop or a reverse-Z shaped hairpin curves and not accompanied by other pancreatic ductal anomaly. Statistical comparison was made among groups on the rate of MMPD including loop and reverse-Z subtypes, MR findings, and clinical features. The rate of MMPD was significantly higher for all idiopathic pancreatitis/idiopathic recurrent acute pancreatitis (RAP) (20%/40%; P<0.001/0.0001; odds ratio (OR), 11.1/29.0) than in the community (2.2%) but was not higher for acute/chronic pancreatitis (14%/8%; P = 0.154/0.266). Multiple logistic regression analysis revealed MMPD to be a significant factor that induces pancreatitis/RAP (P<0.0001/0.0001; OR, 4.01/26.2). Loop/reverse-Z subtypes were found more frequently in idiopathic RAP subgroup (20%/20%; P = 0.009/0.007; OR, 20.2/24.2) than in the community (1.2%/1.0%). The other clinical and radiographic features were shown not associated with the onset of pancreatitis.

**Conclusions:**

MMPD is a common anatomical variant and might be a relevant factor to the onset of idiopathic RAP.

## Introduction

Pancreatitis remains a serious disease and can be fatal in some situations. Causes of pancreatitis include excessive alcohol consumption, biliary stones, autoimmunity, trauma, heredity factors including genetic mutations [1], [2], [3], and several morphological anomalies such as anomalous arrangement of the pancreaticobiliary ductal system (AAPB) [4], [5] or pancreas divisum [5], [6], [7], [8]. However, it is an important task to detect the cause of idiopathic pancreatitis because as many as 20% of cases of pancreatitis [8], [9] and approximately 20–30% of cases of recurrent acute pancreatitis (RAP) [10], [11] remain idiopathic.

The main pancreatic duct (MPD) usually runs smoothly with obtuse-angled curves from the tail and body of the pancreas through the head of the pancreas to the major papilla; in other words, it runs in the antero–posterior, cranio–caudal, and left–right directions. However, we occasionally encounter patients suffering idiopathic pancreatitis, especially idiopathic recurrent acute pancreatitis (IRAP), who have a normal pancreaticobiliary junction but have abnormal curvature in the ventral duct in the head of the pancreas. In those cases, MPD forms a localized spiral or hairpin curve with the appearance of a loop (loop type) or hairpin (reverse-Z type) on coronal projection images of endoscopic retrograde cholangiopancreatography (ERCP) or magnetic resonance cholangiopancreatography (MRCP). Loop type MPD was previously described in a series of AAPB [4] and in a series of pancreas divisum [12], but reverse-Z type MPD was not included in these studies. We grouped and defined these two subtypes as “meandering main pancreatic duct” (MMPD) and we hypothesised that MMPD may contribute in some way to the onset of idiopathic pancreatitis.

To the best of our knowledge, no previous study has investigated the clinical significance of MMPD; however, this topic has long been discussed among Japanese endoscopists as well as pancreas divisum. In the present study, we aimed to determine the unbiased prevalence rate of MMPD in a community population and the effect of MMPD on idiopathic pancreatitis, especially on IRAP, using a non-invasive magnetic resonance (MR) technique.

## Materials and Methods

Based on the Declaration of Helsinki, Research Ethics Committee of the University of Tokyo Hospital approved the prospective and retrospective use of all the corresponding clinical, biochemical, and radiographical data for the present study.

### Subjects

The subjects were divided into two groups and three subgroups. Those in group 1 (Community group) were consecutive subjects in a community population who responded to leaflets and Internet advertising. They participated in a whole-body medical check-up program hosted by our hospital between 12 October 2006 and 31 March 2007, and were enrolled cross-sectionally. The program included a blood test after overnight fasting, whole-body imaging studies including abdominal MR scans and MRCP, evaluation of smoking and drinking habits and medical history, an interview on subjective symptoms, and a physical examination by a board-certified physician. The blood test included white blood cells, haemoglobin, platelets, amylase, C-reactive protein, glycated haemoglobin, glucose, insulin, asparate aminotransferase, alanine aminotransferase, gamma glutamyltransferase, alkaline phosphatase, total bilirubin, high-density lipoprotein, and low-density lipoprotein. All data for each subject were acquired on the same day. Written informed consent for comprehensive epidemiological study was obtained from all subjects. Subjects who underwent the full course of examinations listed above were included into the study.

The subjects in group 2 (Idiopathic pancreatitis group) were a group of patients with idiopathic pancreatitis, which comprised 3 subgroups: (1) idiopathic acute pancreatitis subgroup, (2) idiopathic chronic pancreatitis subgroup, and (3) IRAP subgroup. They were retrospectively extracted from consecutive patients suspected to have any variation of pancreatitis, who visited our hospital between 1 January 2003 and 31 December 2009 and underwent abdominal MR scans including MRCP (patient group). To extract all patients with definitive 3 types of idiopathic pancreatitis from those patients, the entire medical record of each patient was reviewed in detail and types of onset and the cause of pancreatitis was assessed using the latest diagnostic criteria available at the time of March 2010: (1) acute pancreatitis (JPN Guidelines for the management of acute pancreatitis) [13], (2) chronic pancreatitis (The revised Japanese clinical diagnostic criteria for chronic pancreatitis) [14], and (3) RAP (defined as two or more well-documented episodes of abdominal pain, typical of acute pancreatitis, more than 2 months apart and at least one of the following: (i) serum amylase or lipase elevation more than three times the upper limit of normal, (ii) features of acute pancreatitis on imaging [11], [15]). Pancreatitis was diagnosed idiopathic by board-certified gastroenterologists by exclusion of all established causes of pancreatitis, by physical examination, biochemical, and radiographical assessments (genetic and manometric assessments were not done in all cases). Patients with RAP in Idiopathic pancreatitis group belonged to IRAP subgroup. Patients with incomplete evaluations, insufficient MR image quality, post pancreatoduodenectomy state, and neoplasm in the head of pancreas were excluded. Severity of acute and recurrent acute pancreatitis was determined according to the severity scoring system of acute pancreatitis of the Japanese Ministry of Health, Labour, and Welfare (the JPN score 2008) (grade 3–9 was considered severe) [16], contrast-enhanced computed tomography grade (grade 2–3 was considered severe) [16], and non-enhanced computed tomography grade (grade 4–5 was considered severe) [17], [18]), and also to Ranson score (score 3–11 was considered severe) [19] and modified Glasgow score (score 3–8 was considered severe) [20]. Stage of chronic pancreatitis (A, early; B, intermediate; C, end stage) was determined according to a recently proposed criteria [21], [22]. The region of pancreatitis (undetectable; head; body; tail; two or more of these regions) was also recorded [16]. Comprehensive written informed consent for retrospective use of clinical data was obtained from all patients prior to enrolment.

### MR imaging technique

For Community group, MR studies were performed on 3 T scanners (GE Medical Systems, Waukesha, WI). Heavily T2-weighted MRCP images were acquired in the coronal plane by breath-hold two-dimensional half-Fourier fast spin echo (repetition time (TR)/echo time (TE) = ∞/600 ms; slice thickness (ST) = 40 mm) with four coronal and oblique-coronal projection images. For complementary interpretation, transaxial fast spin echo T2-weighted images (TR/TE = ∞/80 ms; ST = 3 mm with no gap) and fat-suppressed T1-weighted images with three-dimensional gradient echo technique (TR/TE = 3.5/1.5 ms; flip angle = 15°; ST = 3 mm with 1.5 mm overlap) were also acquired. No premedication was administered.

For patient group, MR studies were performed on a 3 T scanner (GE Medical Systems) or on a 1.5 T scanner (GE Medical Systems; Siemens AG, Erlangen, Germany; and Toshiba Medical Systems, Tochigi, Japan). Heavily T2-weighted MRCP images were acquired by breath-hold two-dimensional half-Fourier fast spin echo (TR/TE = 2400–∞/600–1100 ms; ST = 30–50 mm) and respiratory-gated three-dimensional half-Fourier fast spin echo (TR/TE = 1300–∞/500–900 ms; ST = 1.2–2.0 mm with no gap), and coronal and oblique-coronal projection images were reconstructed. For complementary interpretation, transaxial and coronal fast spin echo T2-weighted images (TR/TE = 1300–∞/80–150 ms; ST = 5 mm with no gap) and fat-suppressed T1-weighted images with three-dimensional gradient echo technique (TR/TE = 3–840/1.5–140 ms; flip angle = 15°; ST = 1.5 mm with no gap) were also acquired. Manganese chloride solution (Bothdel Oral Solution 10; Kyowa Hakko Kirin, Tokyo, Japan) was administered as negative oral contrast agent prior to MR scanning.

### Image interpretation

All MR images were interpreted independently on picture archiving and communication system workstations (Centricity; GE Medical Systems) by two board-certified diagnostic radiologists with experience in pancreaticobiliary imaging, who were blinded to clinical information. Images determined not to visualize pancreatic ductal anatomy in the head of pancreas clearly by either of the two radiologists were excluded from analyses. Also excluded were images with post pancreatoduodenectomy state or those with neoplasm in the head of the pancreas.

**Figure 1:**
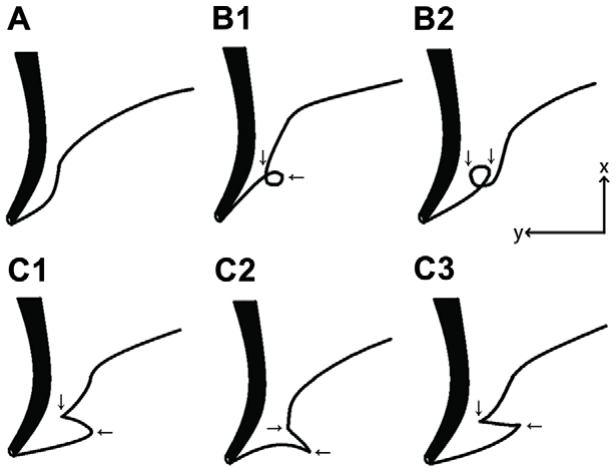
Schematic images of meandering main pancreatic duct (MMPD). The thick line indicates the common bile duct, and the thin line indicates the main pancreatic duct. MMPD was classified into subtypes based on its morphology in the head of pancreas on magnetic resonance cholangiopancreatography: normal type (A), examples of loop type (B1–2), and examples of reverse-Z type (C1–3). Assuming the body-axis as x-axis and horizontal direction as y-axis, MPD curves in loop and reverse-Z types have two extrema in horizontal direction respectively (arrows), while normal type has none. Dorsal pancreatic duct could be observed or not.

The MR data sets were then evaluated for pancreatic ductal anatomy. On (oblique-)coronal planes of MRCP studies, the shape of MPD in the head of pancreas was compared to a mathematical curve assuming a line vertical to the body axis as a y-axis and the body axis as an x-axis (Figure 1). Under this condition, normal type MPD forming a sigmoid curve looks like a cubic curve and seemingly has one inflexion point and has no extremum. MMPD was defined on (oblique-)coronal MRCP plane when (1) located in the head of pancreas; (2) the curve of MPD has two or more extrema in the direction that are vertical to the body axis, forming a curve or angle to make a localized loop (loop type) or reverse-Z shaped hairpins (reverse-Z type); (3) it is not accompanied by AAPB or pancreas divisum (complete or incomplete).

The radiologists were asked to determine if MMPD was present or not and the morphological patterns of MMPD (loop or reverse-Z type) on MRCP images according to its definition and a schema of MMPD (Figure 1) revised from a classification system established previously [12]. Any radiographic findings related to the pancreaticobiliary system were also recorded, if present (e.g., other pancreatic ductal fusion variants, pancreatic cystic lesions, pancreatic ductal/ductile dilatation or irregularity, pancreatic parenchymal atrophy, gallstones, cystic polyps, adenomyomatosis, biliary morphological defects, juxtapapillary duodenal diverticulum). Discrepancies between the two radiologists were settled by the third expert diagnostic radiologist.

### Statistical analysis

Comparison between groups was performed by Student's *t*-test for numerical data and Fisher's exact test for nominal data (univariate analyses). The level of statistical significance was set at 0.05. Family-wise error was corrected by Bonferroni's method. In addition, multiple logistic regression analysis was employed to explore relevant factors for pancreatitis and RAP. To compare the effect of MMPD and pancreas divisum on pancreatitis and RAP, additional multiple logistic regression analysis was made with data including preliminarily excluded patients with pancreas divisum (the same subject group as our previous study [8]). To avoid overestimating the number of predictive values, we selected variables with P<0.05 prior to family-wise error correction at univariate analyses. All statistical computing was performed using the free software R Ver. 2.9 (The R Foundation for Statistical Computing, Vienna, Austria, http://cran.r-project.org/).

## Results

### Subjects

In Community group, 540 subjects fulfilled the whole study, with the following exclusions: incomplete MR scans (n = 1), post pancreatoduodenectomy (n = 1), intraductal pancreatic mucinous neoplasm in the head of the pancreas (n = 3), and insufficient image quality (most commonly gastrointestinal signal hindering visualisation of the pancreatic ducts) (n = 31). The final total of 504 subjects included 205 females (age, 35–84 years; mean, 57.3 years) and 299 males (age, 38–83 years; mean, 56.0 years). One subject was Korean; all others were Japanese. No subject complained of a pancreatic pain.

In patient group, we identified 3,225 MRCP studies from a total of 70,112 MR studies. After excluding patients without pancreatitis, cases of tumor-induced pancreatitis, patients with incomplete evaluation, and overlaps, 237 patients with non-tumor-induced pancreatitis were extracted for analysis (Table 1). After 16 patients with pancreas divisum and one with insufficient image quality (gastrointestinal signal hindering pancreatic ducts) were excluded, we found a final total of 30 cases of definitive idiopathic pancreatitis including 15 females (age, 35–77 years; mean, 54.7 years) and 15 males (age, 24–82 years; mean, 60.4 years), 10 of which were cases of IRAP including 8 females (age, 40–77 years; mean, 54.1 years) and 2 males (age, 63–64 years; mean, 63.5 years) (Table 2, 3, and 4). They were all Japanese.

**Table 1:** Distribution of the causes of non-tumor-induced pancreatitis in patient group.

	Type of pancreatitis			
Cause of pancreatitis	All	Acute	Chronic	Recurrent acute
	(n = 237)	(n = 42)	(n = 166)	(n = 29)
Alcohol	90 [38]	8 [19]	75 [45]	7 [24]
Autoimmunity^a^	52 [22]	2 [5]	49 [30]	1 [3]
Idiopathic	31 [13]	7 [17]	14 [8]	10 [34]
Gallstones	26 [11]	16 [38]	8 [5]	2 [7]
Pancreas divisum^b^	16 [7]	1 [2]	10 [6]	5 [17]
Crohn's disease	3 [1]	1 [2]	2 [1]	0 [0]
Choledochal cyst	3 [1]	3 [7]	0 [0]	0 [0]
Ulcerative colitis treated with salazosulfapyridine	2 [1]	0 [0]	1 [1]	1 [3]
Pancreatic calculus due to IPMN	2 [1]	0 [0]	1 [1]	1 [3]
Hyperlipidemia	2 [1]	1 [2]	0 [0]	1 [3]
Heredity	2 [1]	0 [0]	2 [1]	0 [0]
Alcohol and gallstones combined	2 [1]	0 [0]	2 [1]	0 [0]
Sphincter of Oddi dysfunction	1 [0]	0 [0]	0 [0]	1 [3]
Pancreaticobiliary maljunction	1 [0]	0 [0]	1 [1]	0 [0]
Trauma	1 [0]	1 [2]	0 [0]	0 [0]
Hypothermia	1 [0]	1 [2]	0 [0]	0 [0]
Hypercalcemia	1 [0]	0 [0]	1 [1]	0 [0]
Cholesterol embolism	1 [0]	1 [2]	0 [0]	0 [0]

a ,Diagnosed according to the Asian Diagnostic Criteria of Autoimmune Pancreatitis revised in 2008 [39];

b ,Diagnosed by exclusion [8]; IPMN, intraductal papillary mucinous neoplasm.

Numbers in square brackets represent percentages.

**Table 2:** Clinical features of subjects in Community group with and without meandering main pancreatic duct (MMPD).

	Community group			
	All	MMPD	Non-MMPD	P
	(n = 504)	(n = 11)	(n = 493)	
Age (years) (mean [SD])	56.5 [10.8]	51.4 [11.7]	56.6 [10.1]	0.17^b^
Female (n [%])	205 [40]	4 [36]	201 [40]	1^c^
Brinkman index (cigarettes/day×year) (mean [SD])	314 [459]	171 [300]	317 [461]	0.14^b^
Alcohol intake (kg/year) (mean [SD])	10.6 [17.1]	17.6 [30.7]	10.5 [16.7]	0.31^b^
Clinical history				
Pancreatitis (n [%])	1 [0]	0 [0]	1 [0]	1^c^
Diabetes mellitus (n [%])	30 [6]	1 [9]	29 [6]	0.49^c^
Hypertension (n [%])	79 [16]	0 [0]	79 [16]	0.23^c^
Hyperlipidemia (n [%])	56 [11]	6 [55]	50 [10]	0.004a,c
Any malignant neoplasm (n [%])	25 [5]	0 [0]	25 [5]	1^c^

a ,Significant after family-wise correction;

b ,Student's t-test;

c ,Fisher's exact test; P, P-value for the test between Community group subjects with and without MMPD; SD, standard deviation.

**Table 3:** Clinical features of subjects in Idiopathic pancreatitis group with and without meandering main pancreatic duct (MMPD).

	Idiopathic pancreatitis group				Inter-group
	All	MMPD	Non-MMPD	P_1_	P_2_
	(n = 30)	(n = 6)	(n = 24)		
Age (years) (mean [SD])	57.6 [15.6]	60.7 [9.7]	56.8 [15.6]	0.56^b^	0.73^b^
Female (n [%])	15 [50]	4 [67]	11 [46]	0.66^c^	0.34^c^
Brinkman index (cigarettes/day×year) (mean [SD])	255 [621]	0 [0]	319 [682]	0.031^b^	0.61^b^
Alcohol intake (kg/year) (mean [SD])	5.0 [12.5]	0.2 [14.8]	6.2 [16.4]	0.086^b^	0.059^b^
Clinical history					
Pancreatitis (n [%])	30 [100]	6 [100]	24 [100]	1^c^	<0.0001a,c
Diabetes mellitus (n [%])	3 [10]	0 [0]	3 [13]	1^c^	0.42^c^
Hypertension (n [%])	5 [17]	0 [0]	5 [21]	0.55^c^	1^c^
Hyperlipidemia (n [%])	3 [10]	1 [17]	2 [8]	0.50^c^	1^c^
Any malignant neoplasm (n [%])	2 [7]	1 [17]	1 [4]	0.37^c^	0.66^c^

a ,Significant after family-wise correction;

b ,Student's t-test;

c ,Fisher's exact test; P_1_, P-value for the test between idiopathic pancreatitis patients with and without MMPD; P_2_, P-value for intergroup test between Community group (Table 2) and Idiopathic pancreatitis group; SD, standard deviation.

**Table 4:** Clinical features of subjects in Idiopathic recurrent acute pancreatitis (IRAP) subgroup with and without meandering main pancreatic duct (MMPD).

	IRAP subgroup				Inter-group
	All	MMPD	Non-MMPD	P_1_	P_2_
	(n = 10)	(n = 4)	(n = 6)		
Age (years) (mean [SD])	56 [12.0]	54 [9.9]	57.3 [13.9]	0.67^b^	0.89^b^
Female (n [%])	8 [80]	4 [100]	4 [67]	0.47^c^	0.02^c^
Brinkman index (cigarettes/day×year) (mean [SD])	0 [0]	0 [0]	0 [0]	1^b^	<0.0001a,b
Alcohol intake (kg/year) (mean [SD])	0.2 [0.2]	0.3 [0.3]	0.1 [0.2]	0.36^b^	<0.0001a,b
Clinical history					
Pancreatitis (n [%])	10 [100]	4 [100]	6 [100]	1^c^	<0.0001a,c
Diabetes mellitus (n [%])	0 [0]	0 [0]	0 [0]	1^c^	1^c^
Hypertension (n [%])	1 [10]	0 [0]	1 [17]	1^c^	1^c^
Hyperlipidemia (n [%])	0 [0]	0 [0]	0 [0]	1^c^	0.61^c^
Any malignant neoplasm (n [%])	1 [10]	1 [25]	0 [0]	0.40^c^	0.41^c^

a ,Significant after family-wise correction;

b ,Student's t-test;

c ,Fisher's exact test; P_1_, P-value for the test between IRAP subjects with and without MMPD; P_2_, P-value for intergroup test between Community group (Table 2) and IRAP subgroup; SD, standard deviation.

### Findings and statistical results

MPD in the head of pancreas was clearly visualized in 94.3% (509/540) of Community group and 96.8% (30/31) of Idiopathic pancreatitis group with no significant difference between the groups (Fisher's exact test, P = 0.90).

**Figure 2:**
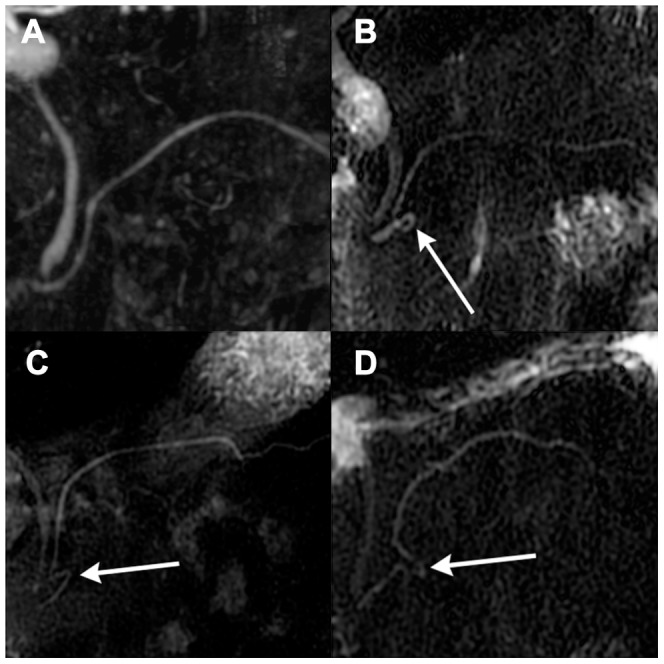
Anatomical variations of meandering main pancreatic duct as seen on magnetic resonance images. (A) Normal type on a magnetic resonance cholangiopancreatography (MRCP) image, (B) loop type on an MRCP image, (C and D) reverse-Z type on MRCP images. The white arrow on subfigures (B, C, D) indicates an abnormally curved section of the main pancreatic duct in the head of pancreas.

In both groups, all MMPD were classified into two categories without ambiguity (no discrepancy was found between the two radiologists): loop type and reverse-Z type (Figure 2). No part of the MPD was shown to run in a postero–anterior direction in any subject.

In Community group, 2.2% (11/504) of subjects had MMPD, including 6 cases of loop type and five of reverse-Z type (Table 5). Pancreas divisum was observed in other 2.6% (13/504) of patients [8] and the others were normal type. A history of hyperlipidemia was more common in subjects with MMPD than without (Table 2). Otherwise, no significant differences were detected in terms of age, gender, clinical history (Table 2), hematologic and biochemical values, or radiographic findings (the only radiographic finding in MMPD subject was 1 adenomyomatosis accompanied by loop type).

**Table 5:** Frequency of meandering main pancreatic duct (MMPD) and its contribution to idiopathic pancreatitis arranged by anatomical subtypes of MMPD and onset types of idiopathic pancreatitis.

	All MMPD^b^	Non-MMPD	P^c^	OR^d^
Community	11 [2]	493		
All idiopathic	6 [20]	24	<0.001^a^	11.1 [3.1–36.2]
Acute	1 [14]	6	0.154	
Chronic	1 [8]	12	0.266	
Recurrent acute	4 [40]	6	<0.0001^a^	29.0 [5.3–144.3]

	Loop type^b^	Else	P^c^	OR^d^
Community	6 [1]	498		
All idiopathic	2 [7]	28	0.069	
Acute	0 [0]	7	1	
Chronic	0 [0]	13	1	
Recurrent acute	2 [20]	8	0.009^a^	20.2 [1.7–139.4]

	Reverse-Z type^b^	Else	P^c^	OR^d^
Community	5 [1]	499		
All idiopathic	4 [13]	26	<0.001^a^	15.1 [2.8–75.0]
Acute	1 [14]	6	0.080	
Chronic	1 [8]	12	0.142	
Recurrent acute	2 [20]	8	0.007^a^	24.2 [2.0–179.2]

a , significant;

b ,numbers in square brackets represent percentages;

c ,Fisher's exact test;

d ,numbers in square brackets represent the 95% confidence interval; OR, odds ratio.

In Idiopathic pancreatitis group, 20.0% (6/30) patients had MMPD, which included two cases of loop type and four of reverse-Z type (Table 5). In IRAP subgroup, 40.0% (4/10) patients had MMPD, which included 2 cases of loop type and 2 of reverse-Z type (Table 5). No MMPD was accompanied by other morphological abnormalities. The others were all normal type. No significant differences in clinical features were detected between subjects with and without MMPD in Idiopathic pancreatitis group (Table 3, P_1_) and in IRAP subgroup (Table 4, P_1_).

In comparing Community group and Idiopathic pancreatitis group, no difference in clinical features was detected except for a higher frequency of pancreatitis in Idiopathic pancreatitis group than in Community group (Table 3, P_2_). However, a higher frequency of pancreatitis, a lower score of Brinkman index, and a lower amount of alcohol consumption were detected in IRAP subgroup than in Community group (Table 4, P_2_). The rate of MMPD and that of loop and reverse-Z types, respectively, were significantly higher in IRAP subgroup than in Community group, with the accompanying odds ratios (ORs) being remarkably high, but these findings did not apply to cases of idiopathic acute/chronic pancreatitis (Table 5).

Multiple logistic regression analysis using MMPD and hyperlipidemia as independent covariates, according to the results of univariate analyses, revealed a significant positive association of MMPD to the onset of pancreatitis (P = 0.0002; OR, 4.01 [95% confidence interval (CI), 1.92–6.11]) and RAP (P<0.0001; OR, 26.2 [95% CI, 22.2–30.2]). Positive association of loop/reverse-Z type to the onset of RAP was also detected (P = 0.0006/0.0009; OR, 21.6/18.5 [95% CI, 15.9–27.3/12.9–24.0]).

Additional multiple logistic regression analysis including preliminarily excluded 16 patients with pancreas divisum revealed a relatively weak association of MMPD to the onset of pancreatitis (P<0.0001; OR, 10.5 [95% CI, 3.57–30.6]) compared to that of pancreas divisum (P<0.0001; OR, 23.6 [95% CI, 10.2–54.4]) and a relatively strong association of MMPD to the onset of RAP (P<0.0001; OR, 21.8 [95% CI, 5.68–84.0]) compared to that of pancreas divisum (P<0.0001; OR, 14.8 [95% CI, 4.37–50.0])

Pancreatitis in patients with MMPD and idiopathic acute pancreatitis or IRAP was possibly less severe and more undetectable or localized in the head of pancreas than pancreatitis in patients without MMPD (Tables 6 and 7); however no significant statistical analysis was available due to insufficient number of data. In patients with idiopathic chronic pancreatitis, the only patient with MMPD had early stage chronic pancreatitis in the head of pancreas, while those without MMPD comprised 7 early, 4 intermediate, and 1 end stage.

**Table 6:** Rate of severe pancreatitis in patients with idiopathic acute and recurrent acute pancreatitis.

Severity index (considered severe)	MMPD	Non-MMPD
CECT score (> = 2) [16]	1/4 [25%]	3/7 [43%]
NECT score (> = 4) [17], [18]	1/4 [25%]	3/9 [33%]
JPN score 2008 (> = 3) [16]	0/5 [0%]	0/11 [0%]
Ranson score (> = 3) [19]	0/5 [0%]	1/11 [9%]
Modified Glasgow score (> = 3) [20]	0/5 [0%]	1/11 [9%]

CECT, contrast enhanced computed tomography; JPN, Japan; MMPD, meandering main pancreatic duct; NECT, non-enhanced computed tomography.

**Table 7:** Involved regions in idiopathic acute and recurrent acute pancreatitis.

Region	MMPD	Non-MMPD
Undetectable	2/4 [50%]	0/10 [0%]
Head	2/4 [50%]	4/10 [40%]
Body	0/4 [0%]	1/10 [10%]
Tail	0/4 [0%]	1/10 [10%]
Two or more regions	0/4 [0%]	4/10 [40%]

MMPD, meandering main pancreatic duct.

## Discussion

This is the first study to focus on the clinical significance of MMPD. We defined MMPD as the MPD forming a loop or a reverse-Z curve in the head of the pancreas in patients with normal pancreaticobiliary junction.We determined the prevalence of MMPD and its subtypes in a community population and in patients with idiopathic pancreatitis using MRCP, and found that the existence of MMPD and its subtypes were significantly associated with the onset of IRAP.

MRCP is a non-invasive diagnostic MR technique that depicts the pancreatic ducts free from radiation exposure, post-procedural pancreatitis, and injection of contrast medium, and has been shown to be highly sensitive and specific (90%–100% for 1.5 T systems) for depicting ventral and dorsal pancreatic ducts [23], [24], [25], [26]. An additional advantage of MRCP is its ability to assess pancreaticobiliary ducts upstream from a proximal obstruction and to depict pseudocysts and mucosal fluid [25]. The disadvantage of MRCP is its limitation in evaluating ampullary lesions [25]. Although secretin-enhanced MRCP is reported to improve pancreatic ductal visualization [27], abnormal responses of MPD to secretin stimulation were previously detected in 12% (8/67) of patients with idiopathic acute pancreatitis including IRAP [27], [28] (anyway, secretin is unavailable in Japan); thus, non-contrasted MRCP alone has been reasonably applicable to the healthy population without clinical indications and to patients in a severe condition such as RAP for the purpose of visualizing pancreatic ductal anatomy. For these reasons, MRCP is routinely performed on patients with pancreatitis of unknown etiology (at the first complete examination or during the follow-up period) to assess the state of the pancreatic ducts and to check for pancreaticobiliary disease in our hospital.

The distribution of the causes of non-tumor-induced pancreatitis including RAP, as obtained in the present study, grossly coincided with that described previously [5], [9], [10], [11], although we observed a slightly lower rate of biliary diseases and higher rate of autoimmune pancreatitis. This could be explained as a result of our hospital being a tertiary referral center and also because autoimmune pancreatitis is a recently established disease and its definition is changing and expanding.

In the present study, MMPD was present in 2.2% of the Community group and in 20.0%/40.0% of patients with idiopathic pancreatitis/IRAP. A previous endoscopy-based study on pancreatic ductal anatomy found loop type MPD in 6.5% (38/585) of a patient group suspected to have pancreaticobiliary disease [12] and 5.4% (2/37) of patients with AAPB [4]. In our other study focusing on alcoholic pancreatitis, only 5.6% (5/90) of patients with alcoholic pancreatitis had MMPD, diagnosed by the same radiologists carefully using the same criteria as in the present study (Gonoi et al. Unpublished). Both these results and those of the present study appear to agree with the hypothesis that MMPD is associated with idiopathic pancreatitis. The rate of MMPD in the Community group was occasionally similar to that of pancreas divisum [8], [29], which is the most common anatomical variant of the pancreas and is found four times as frequently as in European (5.8%) and American (6.0%) population than in Asian population (1.5%) (systematic review) [29]. MMPD might also be found more frequently in European and American population.

Statistical tests comparing Community group to Idiopathic pancreatitis group and IRAP subgroup revealed that MMPD and its subtypes were closely associated with idiopathic pancreatitis and IRAP, supporting the hypothesis of MMPD being a predisposing factor. Lower consumption of alcohol and cigarette in the IRAP subgroup could be because they were told to abstain by their physicians.

In the Community group, history of hyperlipidemia was more common in subjects with MMPD than in those without, while no coincident results were detected in hematologic values probably because they were medicated. No association between hyperlipidemia and MMPD or other pancreatic morphological variations has been reported. However, hyperlipidemia has been reported to cause pancreatitis in pregnancy [30].

Although the present results revealed a strong association between MMPD and pancreatitis, no subject with MMPD in Community group had a history of pancreatitis, radiographic findings, or laboratory data indicative of pancreatitis. We speculate that MMPD may be a predisposing factor for pancreatitis but that small numbers of individuals with MMPD become symptomatic with pancreatitis. This phenomenon is similar to that associated with other pancreatic ductal anomalies; for example, only 5%–10% of patients with pancreas divisum become symptomatic with pancreatitis [28], [31].

The etiology of MMPD is unknown. MMPD is located in the head of the pancreas, where several fusion variations of the ventral and dorsal ducts exist; e.g., AAPB [4], [5], pancreas divisum [5], [6], [7], [8], [23], [24], [25], [31], [32], ansa pancreatica [4], [33], retroportal MPD [34], and other various non-classifiable fusion variants [4], [12], [35], [36], [37]. Of these variants, AAPB [5], pancreas divisum [8], [38], and ansa pancreatica [33], however controversial, have been reported to be associated with pancreatitis and the present results revealed a similar contribution of MMPD and pancreas divisum to the onset of pancreatitis and RAP. Thus, we speculate MMPD to be an analogue of developmental variants that result from abnormal fusion of the ventral and dorsal anlagen of the pancreas in the fetal stage, rather than a pancreatic ductal irregularity caused by pancreatitis.

The mechanism by which MMPD is associated with RAP is not yet elucidated. As we observed no MPD and dorsal pancreatic ductal dilatation or pancreatic parenchymal atrophy associated with MMPD, mechanical obstruction theory as proposed in pancreas divisum seems less conceivable [25], [28], [31]; however, in the present study, the only specified region of pancreatitis in cases with MMPD was the head of pancreas, and after the present study series, we have experienced a single case of reverse-Z type accompanied by Wirsungocele, which may support the mechanical obstruction theory. Otherwise, some genetic etiologies could be accompanied by the presence of MMPD; like as cystic fibrosis transmembrane conductance regulator gene (CFTR) being found frequently in patients with pancreas divisum and RAP [1], [38]. Alternatively, some genetic mutations, such as CTFR [3] or serine protease inhibitor Kazal type 1 [1], [2] themselves might be the causative factor for RAP in the presence of MMPD. Further investigation is needed.

The major limitation in the present study is that the definition of MMPD has some arbitrariness. Although it might be less practical, more strict three-dimensional and mathematical analysis could be performed in the future study. Minor limitations are as follows. First, the subjects in Community group were not randomly chosen from the community and might be more interested in health. Second, the MR scans in Idiopathic pancreatitis group were acquired by non-identical, but quite similar, settings. Third, in some cases, MRCP might have failed to visualize a thin looped type MPD and misclassified it into normal type due to limited spatial resolution. Fourth, the present study was a cross-sectional study and we did not show experimental or prospective evidence that MMPD causes pancreatitis as well as pathological assessment. In a strict sense, a long-term follow-up study is required that includes a large number of individuals with MMPD, from a young age. Finally, as our study group included small numbers of IRAP patients, a next step would be to evaluate pancreatic ductal anatomy in a larger group of IRAP, which would require a multicenter collaborative study.

In conclusion, this is the first study to focus on the clinical significance of MMPD. We revealed that MMPD is a relatively common variation of the pancreatic ductal anatomy and that it is found highly frequently in patients with IRAP. We conclude that MMPD might be considered a relevant factor to the onset of IRAP.
